# Neuroprotective Effect of *Cudrania tricuspidata* Fruit Extracts on Scopolamine-Induced Learning and Memory Impairment

**DOI:** 10.3390/ijms21239202

**Published:** 2020-12-02

**Authors:** Seung-Cheol Jee, Kwang Min Lee, Min Kim, Yoo-Jung Lee, Soee Kim, Joon-Oh Park, Jung-Suk Sung

**Affiliations:** Department of Life Science, Biomedi Campus, Dongguk University-Seoul, 32 Dongguk-ro, Ilsandong-gu, Goyang-si 10326, Gyeonggi-do, Korea; markjee@naver.com (S.-C.J.); lkm5411@hanmail.net (K.M.L.); pipikimmin@naver.com (M.K.); wjdyd85@naver.com (Y.-J.L.); soeesoee@naver.com (S.K.); joonoh5@dongguk.edu (J.-O.P.)

**Keywords:** *Cudrania tricuspidata*, scopolamine, CREB, ERK1/2, learning and memory

## Abstract

*Cudrania tricuspidata* has diverse biological activities, such as antioxidant, anti-inflammatory, anticancer, and neuroprotective effects. This study investigated the protective effects of *C. tricuspidata* fruit extracts (CTFE) against scopolamine (SCO)-induced neuron impairment. The neuroprotective effects of CTFE on SCO-induced memory dysfunction were confirmed in mice using the Barnes maze test. The results showed that co-treatment of SCO and CTFE increased the stay time in the target zone compared with SCO treatment alone. Similarly, the results obtained by the fear conditioning test revealed that SCO-CTFE co-treatment induced the freezing action time under both the contextual fear condition and the cued fear condition compared with SCO treatment alone. Moreover, we showed that CTFE reduced the SCO-induced acetylcholinesterase (AChE) activity, thereby increasing the acetylcholine concentration in mice hippocampal tissues. Consistent with the improvement of memory and recognition function in vivo, our in vitro results showed that CTFE induced cAMP response element binding protein (CREB) and extracellular regulated kinase 1/2 (ERK1/2) activity in PC12 cells and reduced SCO-induced AChE activity. In addition, the microarray results of the hippocampal tissue support our data showing that CTFE affects gene expressions associated with neurogenesis and neuronal cell differentiation markers such as *spp1* and *klk6*. Overall, CTFE exerts a neuroprotective effect via regulation of the CREB and ERK1/2 signaling pathways and could be a therapeutic candidate for neurodegenerative diseases.

## 1. Introduction

The world’s population is aging, leading to the development of degenerative brain disease. Age-related degenerative brain diseases include Parkinson’s disease, Alzheimer’s disease (AD), Huntington’s disease, amyotrophic lateral sclerosis, and multiple sclerosis [1,2]. Degenerative brain diseases impair learning and memory, deteriorating the patient’s quality of life and affecting the lives of family caregivers and society [3,4]. Generally, cholinergic drugs can be used for degenerative brain diseases, such as AD, by inhibiting acetylcholinesterase (AChE) or modulating muscarinic acetylcholine receptors (mAChRs) and nicotinic acetylcholine receptors [5]. However, because of the short half-life, the improvements in learning and memory are only temporary, and the drug must be taken several times a day [6]. In addition, cholinergic drugs can cause head pressure, ataxia, vomiting, renal failure, and other side effects [7].

Acetylcholine (ACh) is a neurotransmitter that plays an important role in learning and memory [8]. ACh is synthesized by choline acetyltransferase (ChAT), which catalyzes the acetylation of choline with acetyl-CoA [9]. ACh is released from presynaptic terminals in vesicles and diffuses toward postsynaptic terminals. Its signaling-mediated cell surface receptors (nAChRs and mAChRs) are bound tothe surface of cells. nAChRs are ion channels composed of five subunits [10]. mAChRs are single polypeptide proteins with seven transmembrane domains that evoke intracellular responses by interacting with heterotrimeric G proteins [10].

Scopolamine (SCO) is a tropane alkaloid that is mainly used in preclinical experiments to induce amnesia in animals. Although it is a non-selective mAChR antagonist, it shows high selectivity for mAChR, and high doses of SCO can block nAChRs [11]. SCO-induced memory impairment can appear in human subjects [12]. Moreover, SCO is considered the ‘gold standard’ drug that causes experimental memory impairment [13].

*Cudrania tricuspidata* (*C. tricuspidata*), a deciduous broad-leaved thorn tree, belongs to the family Moraceae and is widespread in Korea, Japan, and China [14]. *C. tricuspidata* is a well-known herb in Oriental medicine, of which the root, leaf, and fruit are frequently used in traditional medicine [15]. The *C. tricuspidata* fruit contains 158 flavonoids and 99 xanthones and has multiple biological activities, such as antioxidant, anti-inflammatory, immunomodulatory, and monoamine oxidase inhibitory activities [16,17]. A recent study demonstrated the neuroprotective activity of *C. tricuspidata* fruit extracts (CTFEs) against 6-hydroxydopamine-induced oxidative stress in vitro [18].

cAMP response element binding protein (CREB) is a major transcription factor that regulates the gene expression related to neurogenesis, neuroprotection, and synaptic activity [19]. CREB is known to be involved in spatial, fear, olfactory, and object and social recognition memory [20]. Previous studies on AD showed that β-amyloid induced pathogenesis through the CREB pathway, and CREB expression decreased in patients’ brains [21,22]. Upstream of CREB, extracellular regulated kinase 1/2 (ERK1/2) regulates the neuronal function and development of the central nervous system (CNS) and is related to memory formation [23,24]. Many studies thereby target the ERK1/2-CREB signaling pathway for developing treatments for degenerative diseases such as AD [25]. AMP-activated protein kinase (AMPK), also upstream of CREB, is associated with brain injury and neurological disorders, including down-regulation in AD [26,27]. Moreover, there is a link between sirtuin-3 (SIRT3) and neurodegenerative diseases in relation to AMPK [28,29]. A study showed that SIRT3 protects neurons from ischemia by inducing autophagy through AMPK activation [30].

Here, we hypothesized that CTFE would aid learning and memory function by exerting its diverse abilities, like neuroprotection. The strategy involved in vitro experiments using the rat pheochromocytoma-derived cell line (PC12), and animal behavioral tests to confirm improvements and the overcoming of learning and memory dysfunction caused by SCO. In addition, in order to evaluate alterations in cholinergic markers (ACh and AChE) and elucidate whether intracellular signaling pathways and genomic expression are affected, male ICR mice were sacrificed to obtain hippocampal tissue.

## 2. Results

### 2.1. Cytotoxicity of SCO and CTFE on PC12 Cells

**Figure 1:**
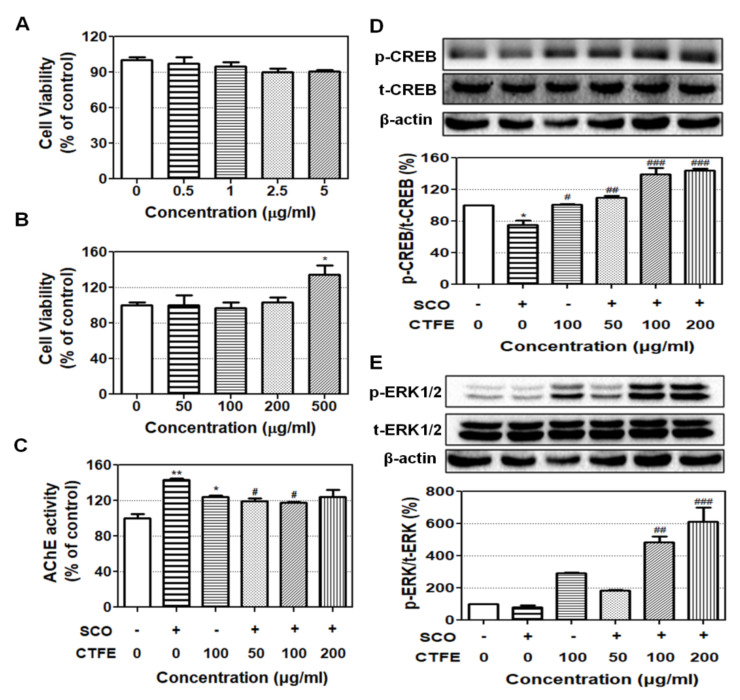
Effects of SCO and CTFE on PC12 cells. Cell viability when treated with various concentrations of (**A**) SCO and (**B**) CTFE. (**C**) The inhibitory effect of CTFE on SCO-induced acetylcholinesterase (AChE) activity was measured. The activation of (**D**) cAMP response element binding protein (CREB) and (**E**) extracellular regulated kinase 1/2 (ERK1/2) was evaluated. All data were expressed as the mean ± SEM (*n* = 3) * *p* < 0.05 and ** *p* < 0.01 vs. control. ^#^ *p* < 0.05, ^#^^#^ *p* < 0.01, and ^#^^##^ *p* < 0.001 vs. SCO group. SCO: scopolamine, CTFE: *Cudrania tricuspidata* fruit extract, GAL: galantamine.

To confirm the cytotoxicity of SCO and CTFE, differentiated PC12 cells were exposed to SCO and CTFE at various concentrations for 24 h. As shown in Figure 1A, treatment of SCO at 0.5, 1.0, 2.5, and 5.0 µM for 24 h showed no cytotoxicity. We chose the concentration of SCO (2.5 µg/mL) for further experiments. CTFE did not induce cytotoxicity in PC12 cells, but 500 µg/mL CTFE treatment significantly increased cell viability by over 30% compared with the other concentrations (Figure 1B). Our results confirm that CTFE has no cytotoxicity, and concentrations of 50, 100, and 200 µg/mL CTFE can be used for further experiments.

### 2.2. AChE Inhibitory Effect of CTFE on PC12 Cells

To assess the AChE activity in PC12 cells, we performed an AChE activity assay. As Figure 1C illustrates, when PC12 cells were exposed to SCO, the AChE activity was significantly increased compared with the control group. Treatment of 100 µg/mL CTFE also increased the AChE activity compared with the control. Conversely, SCO+ 50, 100, and 200 µg/mL CTFE significantly attenuated the AChE activity level compared with the SCO group. These results suggest that CTFE attenuates the SCO-induced AChE activity level.

### 2.3. Effect of CTFE on Protein Levels of CREB and ERK1/2 in PC12 Cells

The CREB and ERK1/2 signaling pathways are well known to be involved in neuroprotection [31,32]. To evaluate the neuroprotective effects of CTFE, the CREB and ERK1/2 pathways were analyzed in PC12 cells by Western blot. As a result, there was no change in the expression of total CREB and ERK1/2, but the phosphorylated forms differed from the control (Figure 1D,E). The p-CREB and p-ERK1/2 levels were decreased in the SCO group but increased in the SCO + 50, 100, and 200 µg/mL CTFE groups. These results indicate that CTFE highly activates CREB and ERK1/2 against SCO-induced damage.

### 2.4. Effect of CTFE on SCO-Induced Learning and Memory Dysfunction of Mice in BMT

**Figure 2:**
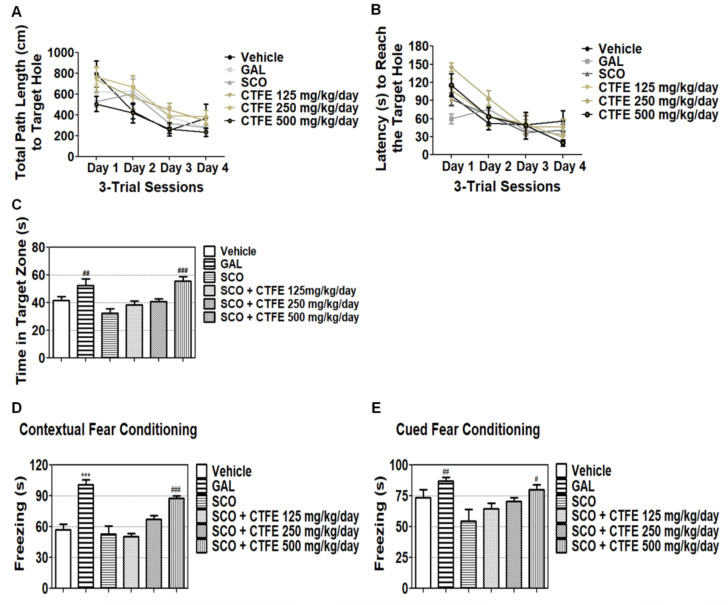
CTFE enhances spatial and fear learning and memory function in the behavioral test. In the Barnes maze test, (**A**) movement distance and (**B**) escape latency were measured during the training phase. (**C**) In the probe phase, the duration time in the target zone was evaluated. In the fear conditioning test, the effect of SCO and CTFE treatment on freezing time of the (**D**) contextual and (**E**) cued fear conditioning test was measured. All data were expressed as the mean ± SEM (*n* = 7). *** *p* < 0.001 vs. vehicle group. ^#^ *p* < 0.05, ^##^ *p* < 0.01, and ^###^ *p* < 0.001 vs. SCO group. GAL: galantamine, SCO: scopolamine, CTFE: *Cudrania tricuspidata* fruit extract.

The BMT was undertaken to assess the improvement in spatial memory capacity. No drugs were injected during the training phases, thereby equalizing the level of spatial learning. During a probe phase, each group was injected with each drug. In the last training phase, the distance (Figure 2A) and latency time (Figure 2B) of all groups reaching the escape box were similar to each other. These results indicate that all groups had learned to use the spatial cues required to locate the escape box, and we considered that all groups had a similar level of spatial learning and memory.

To evaluate the spatial memory, the escape box was removed, and mice were observed for 90 s. During the probe phase (Figure 2C), the stay time was reduced slightly for the SCO group relative to the vehicle group, which was similar to co-treatment of SCO with 125 and 250 mg/kg/day CTFE. GAL (positive control) and the SCO + 500 mg/kg/day CTFE group remained longer, for 52.55 ± 4.56 and 55.53 ± 3.46 s, respectively, compared with the SCO group (32.64 ± 3.14 s; ^##^ *p* < 0.01 and ^###^ *p* < 0.001 vs. SCO group). These results indicate that SCO decreases spatial memory capacity, but CTFE inhibits SCO-induced spatial memory impairment and activates spatial memory.

### 2.5. Effect of CTFE on SCO-Induced Learning and Memory Dysfunction of Mice in the FCT

Fear learning and memory were evaluated by the FCT. We analyzed contextual and cued fear separately. In both tests (Figure 2D), freezing time was increased in the GAL (positive control) and the SCO + 500 mg/kg/day CTFE group (contextual fear: 100.97 ± 4.61 and 87.58 ± 2.70 s, respectively; ** *p* < 0.01 and *** *p* < 0.001 vs. vehicle group, ^###^ *p* < 0.001 vs. SCO group; cued test: ^#^ *p* < 0.05 and ^##^ *p* < 0.01 vs. SCO group). In the cued FCT (Figure 2E), freezing time was increased by CTFE in a dose-dependent manner. These results suggest that CTFE increases fear learning and memory by inhibiting SCO, and a high dose of CTFE increases these aspects dramatically.

### 2.6. Effect of CTFE on ACh Concentration and AChE Activity in Mice

**Figure 3:**
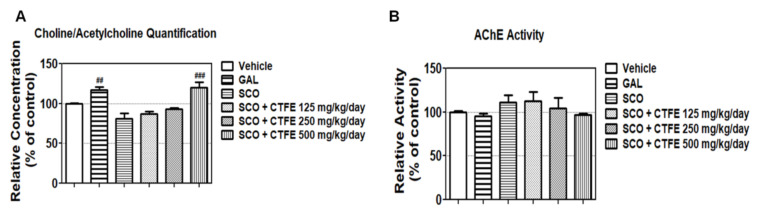
Effects of CTFE on ACh concentration in blood and AChE activity in hippocampal tissue. (**A**) Relative quantitative evaluation of ACh in blood. (**B**) AChE activity in hippocampal tissue. All data were expressed as the mean ± SEM (*n* = 3). ^##^ *p* < 0.01 and ^###^ *p* < 0.001 vs. SCO group. ACh: acetylcholine, AChE: acetylcholinesterase, GAL: galantamine, SCO: scopolamine, CTFE: *Cudrania tricuspidata* fruit extract.

Quantification of the ACh concentration in blood (Figure 3A) revealed that SCO decreased the relative concentration of ACh by 81.34 ± 6.72%, but the GAL (positive control) and the SCO+ 500 mg/kg/day CTFE treatments increased the ACh concentration compared with the SCO group (^##^ *p* < 0.01 and ^###^ *p* < 0.001 vs. SCO group). As shown in Figure 3B, the AChE activity in hippocampal tissue was slightly decreased in the GAL (positive control) and the SCO + 500 mg/kg/day CTFE group (95.01 ± 3.11% and 97.20 ± 1.11%, respectively) relative to the SCO group. These results suggest that CTFE can affect learning and memory by increasing the ACh concentration in blood and decreasing the AChE activity.

### 2.7. Effect of CTFE on Protein Levels of SIRT3 and AMPK in Hippocampal Tissue of Mice

**Figure 4:**
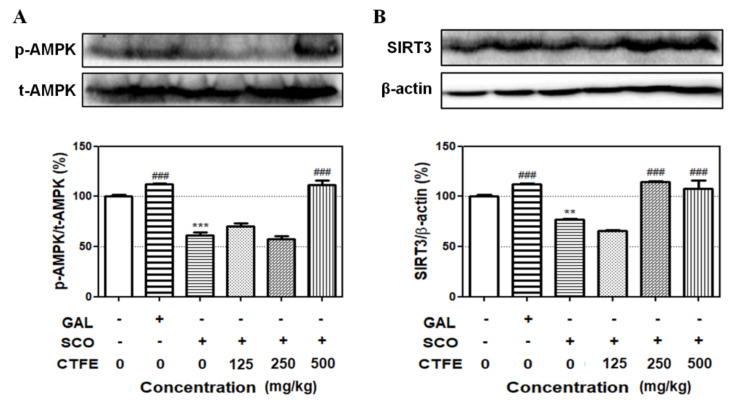
Effects of SCO and CTFE on activation of AMPK and expression of SIRT3 in hippocampal tissue. (**A**) The activation of AMPK and (**B**) expression of SIRT3 were evaluated. All data were expressed as the mean ± SEM (*n* = 3) ** *p* < 0.01 and *** *p* < 0.001 vs. control. ^#^^##^ *p* < 0.001 vs. SCO group. SCO: scopolamine, CTFE: *Cudrania tricuspidata* fruit extract, GAL: galantamine.

The AMPK and SIRT3 signaling pathways are associated with learning and memory [33,34]. To evaluate the effects of CTFE on learning and memory, the activation of AMPK and SIRT3 was analyzed in hippocampal tissue by Western blot. While the total level of AMPK was not affected, CTFE induced the phosphorylated forms compared to the SCO treatment group (Figure 4A). SCO significantly reduced the SIRT3 expression compared to the non-treatment group, but co-treatment of SCO with 250 and 500 mg/kg of CTFE significantly increased the SIRT3 expression (Figure 4B). These results indicate that CTFE induces AMPK activity and SIRT3 expression against SCO-induced cognitive impairments.

### 2.8. Global Gene Expression in Hippocampal Tissue of Mice

**Figure 5:**
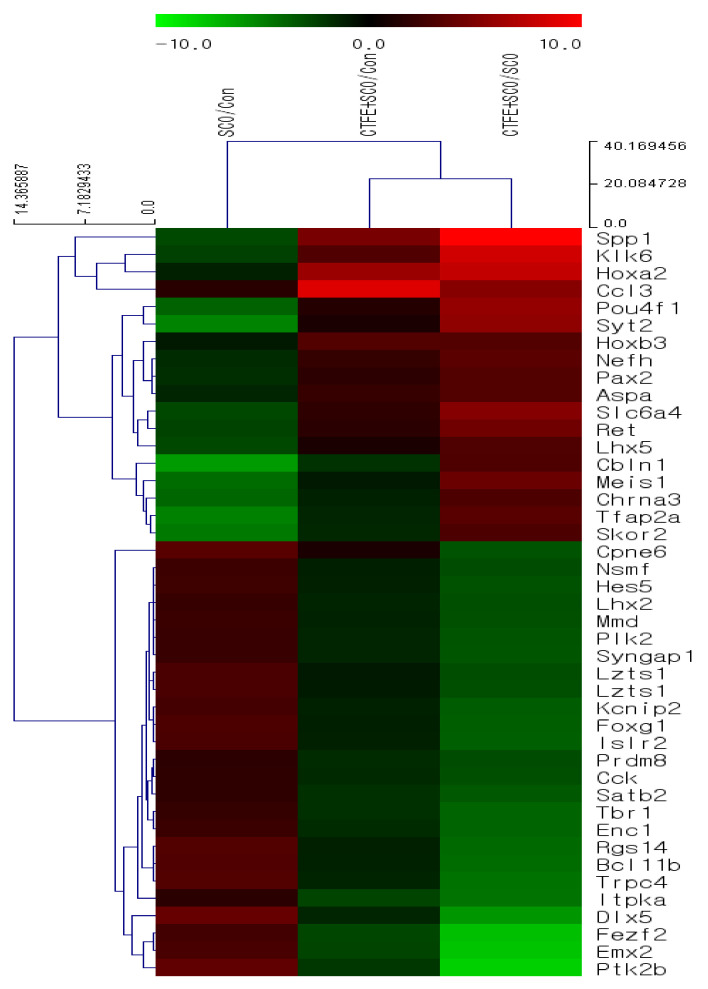
The list of selected genes shows the greatest up-regulated fold changes (>3) in the SCO + CTFE group compared to the SCO group using hierarchical clustering analysis of MeV program. Fold change was calculated as log2 ratio between SCO + CTFE group and SCO group. All results were calculated after setting a normalized value (log2) of 4.00 and a *p*-value of 0.05.

The microarray analysis indicated that SCO treatment alone decreased the expression of genes associated with neurogenesis and neuronal cell differentiation, such as *secreted phosphoprotein1* (*spp1*) and *kallikrein-related peptidase 6* (*klk6*) compared with the vehicle group (Figure 5). By contrast, SCO + CTFE treatment marginally and dramatically (3- to 21-fold) increased the gene expressions compared with the vehicle group and SCO group, respectively. These results indicate that CTFE activates neurogenesis and neuronal cell differentiation by inhibiting SCO-induced down-regulation of gene expressions.

## 3. Discussion

In the present study, we investigated the neuroprotective effects of CTFE on PC12 cells and SCO-induced mice amnesia models. Before the test, we evaluated the neuroprotective effect of CTFE against SCO-induced damage in PC12 cells. PC12 cells are neuron-like rat pheochromocytoma cells that possess mAChR are sensitive to SCO, a negative control that induces cognitive and memory impairment, as well as amnesia [35]. Our results confirmed that up to 5 and 500 mg/mL of SCO and CTFE, respectively, were not cytotoxic for PC12 cells (Figure 1A,B).

ACh is used as a marker for the activation of neuronal function [36]. It is well recognized that cholinergic drug injections exert robust effects on learning and memory, and the pharmacological effects on learning and memory are closely associated with ACh release in the hippocampus, striatum, and amygdala [37]. Moreover, ACh is rapidly degraded by AChE, thereby terminating the signal transmission. Numerous studies suggest that AChE is a biomarker of neurotoxicity [38]. Our results showed that SCO induced AChE activity, whereas co-treatment of SCO + CTFE significantly reduced the AChE activity compared with SCO alone in our in vitro model (Figure 1C). On the other hand, AChE activity was increased by CTFE treatment compared to the untreated control group (Figure 1C). AChE has three variants known as AChE-S, AChE-R, and AChE-E and they have multiple biological effects [39]. Previous studies showed that AChE was related to neuronal differentiation and mineralization, as well as extension of neurite outgrowth [40,41]. In addition, AChE activity is not limited to cholinergic transmission and is associated with several non-cholinergic actions such as cell proliferation [42]. These results indicate that moderately increased AChE activity does not always have negative effects involved in degenerative diseases. Therefore, our results suggest that CTFE acts as an inhibitor of SCO and possesses neuroprotective effects against degenerative brain disease.

GAL is a reversible, competitive AChE inhibitor that was used in this study as a positive control [43]. GAL is used as an AChE drug treatment for AD, which has been accepted in many countries and received Food and Drug Administration (FDA) approval for the treatment of mild-to-moderate AD in 2001 [44]. GAL acts as a neuroprotective agent, as indicated by its capacity for defending against the cytotoxic effects of glutamate, hypoxia resulting from trophic factor deprivation, and amyloid-beta neurotoxicity. In addition, GAL enhances neurotransmitter release and improves memory performance in animal models [45]. Therefore, we used GAL as a positive control and SCO as a negative control in animal studies.

Based on the in vitro results, we conducted the BMT and FCT to confirm the neuroprotective effect of CTFE on rats. The Barnes maze was developed by Carol Barnes to overcome the stress induced by swimming in the Morris water maze [46]. It is commonly used to evaluate spatial learning and memory in mice based on whether they effectively solve a maze using multiple strategies (e.g., target location, spatial cues, or cue condition). When tested for spatial learning tasks using the escape box, to confirm that similar levels of proficiency have been achieved between all mice looking for an escape box, all mice were not injected with drugs for days 1–4. Until day 4, the number of errors, the time of latency to enter the escape box, and the total distance to enter the escape box were recorded. Our results confirmed that a similar level of proficiency was acquired between all mice looking for an escape box (Figure 2A,B). On day 5 (probe phase), SCO + 125, 250, and 500 mg/kg/day CTFE groups spent more time in the target zone than the SCO group in a dose-dependent manner (Figure 2C). A previous study has shown that SCO is known to impair spatial working memory [47]. The result supports the fact that the SCO group’s reduced duration time indicates memory retrieval impairment. Our results suggest that SCO inhibits spatial memory function, whereas CTFE improves spatial memory by increasing memory retrieval ability. Moreover, tests for contextual and cued fear are used to evaluate the ability of mice to learn and remember an association between environmental cues and aversive experiences [48]. We confirmed that the SCO group had a shorter freezing time than the 500 mg/kg/day CTFE group in the contextual and cued FCT (Figure 2D,E). These results suggest that CTFE has a protective effect against SCO-induced memory impairment.

ChAT synthesizes ACh by using substrates such as choline and acetyl-CoA [9]. Choline is synthesized in the liver, some of which is derived through dietary intake, and is transferred across the blood–brain barrier through specific membrane transporters [49]. Moreover, the levels of choline and ACh in the blood are associated with brain ACh concentration [50]. To confirm the regulation effect of ACh levels and AChE activity by CTFE, blood was collected from micebefore sacrifice. Furthermore, to prevent other factors, we used a small amount of ether to anesthetize mice to prevent changes in choline/Ach caused by anesthetic [49]. Our result confirmed that CTFE increases ACh levels compared to SCO (Figure 3A). A previous study supported the fact that large amounts of ACh were found to prevent SCO-induced memory dysfunction [51]. Moreover, GAL (positive control) and the SCO + 500 mg/kg/day CTFE group displayed slightly reduced AChE activity compared to the SCO group (Figure 3B). These results indicate that CTFE inhibits ACh removal by SCO, thereby enhancing cholinergic neurotransmission [52]. On the other hand, a previous study showed that AChE inhibition induces side effects dependent on the inhibition rate of AChE activity [38]. Our results confirmed that the inhibition rate of AChE (within 10%) by CTFE is similar to the GAL treatment group (within 10%). Overall, our results suggest that CTFE regulates choline and ACh levels, and improves choline function, as it has a blood–brain barrier penetration ability. Moreover, CTFE is a safe natural material with a similar efficacy to GAL; therefore, it can be considered as a candidate for treatment of degenerative brain disease.

To investigate the intracellular signaling pathway involved in learning and memory, we selected CREB, ERK1/2, AMPK, and SIRT3. Previous studies have shown that these proteins are associated with neuronal survival and are activated by each other [31,32]. CREB regulates the expression of genes related to brain-derived neurotrophic factor, which promotes neuronal survival, memory consolidation, and synaptic plasticity [53]. Activation of CREB is required for the formation and retention of memory, which is the main physiological parameter of AD [54]. Moreover, reduced p-CREB has been observed in the postmortem brains of patients with AD [55]. ERK1/2 is a mitogen-activated protein kinase (MAPK) with a critical role in learning and memory. Previous studies have shown that its signaling is required for the activation of transcription during long-term memory and short-term memory formation [56,57]. ERK1/2 coordinates neuronal responses to extracellular signals and affects cell growth, differentiation, migration, and proliferation [58]. It is also known that ERK1/2 regulates synaptic remodeling, axonal growth, long-term potentiation, and neuronal excitability [59]. Our results demonstrate that although total CREB expression is comparable to ERK1/2 expression, the phosphorylated forms of CREB and ERK1/2 increase in the SCO + CTFE groups compared with SCO treatment alone (Figure 1D,E). The activation of CREB depends on phosphorylation by AMPK [60]. Moreover, AMPK is essential for the regulation of neuronal energy metabolic plasticity related to synaptic activation, which causes cognitive impairment [61]. Furthermore, a close association between AMPK and SIRT3 was indicated from a previous study showing that ischemic brain injury was protected through a positive feedback loop between two molecules [62]. We confirmed that CTFE induces AMPK activation and SIRT3 expression compared to the SCO treatment group (Figure 4). A study showed that autophagy was essential for anti-inflammatory and synaptic plasticity in glial cells [63]. Autophagy has an important role in cell survival in neuronal cells and elimination of abnormal protein. Moreover, autophagy was down-regulated in neurodegenerative diseases in accordance with the regulation of ERK [64,65]. AMPK, which is upstream of ERK in autophagy, induces autophagy activation [66]. Another study confirmed that chebulagic acid induces neuroprotective effect by inducing autophagy [67]. These results suggest that CTFE protects against SCO-induced neuronal impairment by inducing autophagy through regulation of SIRT3, AMPK, ERK1/2, and CREB signaling pathways.

To confirm the neuroprotective effect of CTFE, we performed microarray analysis on the mice hippocampal tissues. Our data showed that CTFE increases the expression of *spp1*, *klk6*, and other genes related to neurogenesis and neuronal cell differentiation based on the DAVID database (Figure 5). SPP1 (also called osteopontin) is known to play an important role in postischemic lateral migration of neuroblasts and tissue remodeling following cerebral ischemia [68]. Induction of SPP1 enhanced neuroprotective effects by regulating neuroinflammation markers such as nitric oxide synthase and nitric oxide in microglia [69]. Other research showed high SPP1 expression in the mild cognitive impairment group compared to the untreated control and dementia groups [70]. KLK6 serves various physiological functions in the central nervous system, including cell differentiation and survival by activating the components of the MAPK and AKT signaling cascades in central nervous system-derived cells [71]. Previous studies showed that KLK6 was related to neuroinflammation and neurodegenerative diseases, as it was induced in the mild cognitive impairment group compared to the dementia group [70,72]. In association with cognitive deficits, by regulating large-scale brain networks, neuroinflammation is indicated to be correlated with the progression of AD [73]. Thus, CTFE is suggested to have a neuroprotective effect on SCO-induced cognitive impairment by regulating autophagy in neuroinflammation through SIRT3-AMPK/ERK1/2-CREB signaling pathways. Overall, CTFE affects neurogenesis and neuronal cell differentiation, as well as synaptic plasticity, memory consolidation, and other neuronal functions. Improvements in behavioral expression, cholinergic function, and the cholinergic signaling pathway were thought to result from the inhibitory effect of CTFE on alterations in SCO-induced gene expression. Based on our results, among the numerous chemicals in CTFE, the candidates for neuroprotective effects were expected to be at least one of the following four chemicals: 4′-*O*-methylalpinumisoflavone, 6,8-diprenyl orobol, 6,8-diprenyl genistein, oralpinumisoflavone.

In conclusion, SCO blocks the stimulation of postsynaptic acetylcholine receptors [13] and induces learning and memory dysfunction. CTFE improves cholinergic function by reducing AChE activity and increasing the ACh concentration against SCO-induced damage. Furthermore, SCO inhibits the activation of SIRT3, AMPK, CREB and ERK1/2, causing learning and memory dysfunction, but CTFE can recover it. In addition, CTFE affects gene expressions associated with neurogenesis and neuronal cell differentiation. Our results suggest that CTFE has a therapeutic effect on neurodegenerative diseases related to learning and memory dysfunction by improving cholinergic systems and regulating intracellular signaling and gene expression.

## 4. Materials and Methods

### 4.1. Chemicals and Reagents

(−)-SCO methyl bromide and galantamine (GAL) hydrobromide were purchased from Sigma-Aldrich (St. Louis, MO, USA). SCO was dissolved in 0.9% saline solution for animal administration. Roswell Park Memorial Institute (RPMI) 1640 medium was purchased from Welgene (Daegu, Korea). Horse serum (HS), fetal bovine serum (FBS), and penicillin/streptomycin (P/S) were purchased from Gibco/Life Technologies (Breda, The Netherlands).

### 4.2. Plant Extract

**Figure 6:**
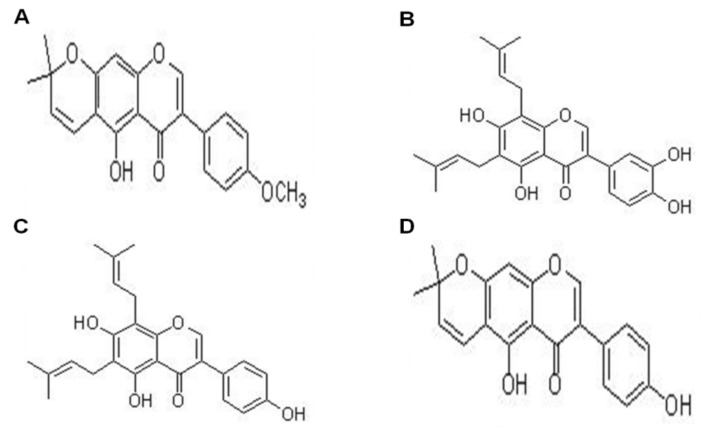
The structure of chemicals in *Cudrania tricuspidata*. (**A**) 4′-*O*-methylalpinumisoflavone, (**B**) 6,8-diprenyl orobol, (**C**) 6,8-diprenyl genistein, and (**D**) alpinumisoflavone.

*Cudrania tricuspidata* fruit was purchased from Hampyeong Farming Association (Jeollabuk-do, Korea). After drying the fruit (100 kg) using a hot-air dryer at 40 °C for 72 h, 20 kg of dried *C. tricuspidata* fruit was obtained. Dried *C. tricuspidata* fruit was extracted in a high-speed vacuum low-temperature extractor (Cosmos, Incheon, Korea) with 10-fold 50% methanol at 80 °C for 4 h and then heated in boiling water at 100 °C. After freeze-drying, CTFE was kept in a 4 °C chamber. A previous study has shown that 4′-*O*-methylalpinumisoflavone, 6,8-diprenyl orobol, 6,8-diprenyl genistein, and alpinumisoflavone were identified in CTFE (Figure 6) [74].

### 4.3. Cell Culture

The PC12 cell line was obtained from the American Type Culture Collection (ATCC) Global Bioresource Center (Manassas, VA, USA). The undifferentiated PC12 cells were cultured in a growth medium (RPMI 1640) supplemented with 5% FBS, 10% HS, and 1% P/S at 37 °C in a humidified atmosphere of 5% CO_2_. For nerve growth factor (NGF)-induced differentiation studies, PC12 cells were cultured in differentiating medium (RPMI 1640) supplemented with 1 µg/mL NGF, 1% FBS, 0.5% HS, and 1% P/S at 37 °C in a humidified atmosphere of 5% CO_2_.

### 4.4. Cell Viability Assay

NGF-induced differentiated PC12 cells were seeded in differentiating medium at 10 × 10^3^ cells/well in a 96-well microplate for assessing SCO and CTFE cytotoxicity by the WST-1-based method. After exposure to different concentrations of CTFE (0, 50, 100, 200, and 500 µg/mL) and SCO (0.0, 0.5, 1.0, 2.5, and 5.0 µg/mL) for 24 h, the cells were incubated with 110 µL of differentiating medium containing 10% EZ-Cytox Reagent (Daeil Lab Service, Seoul, Korea) at 37 °C for 2 h. Afterward, absorbance was measured at 450 nm using a microplate ELISA reader (TECAN, Männedorf, Switzerland).

### 4.5. AChE Activity Assay

To assess the effect of CTFE on AChE, the differentiated PC12 cells were seeded in differentiating medium at 10^4^ cells/well in a 96-well microplate for assaying the AChE activity. The cells were exposed to 2.5 µg/mL SCO and different concentrations of CTFE (50, 100, and 200 µg/mL) for 24 h. Afterward, the AChE activity was measured at 37 °C for 10–30 min using an AChE Colorimetric Assay Kit (BioVision, Mountain View, CA, USA). Absorbance at 570 nm was measured in a microplate ELISA reader (TECAN). The effects of SCO and CTFE on AChE activity were determined by comparing the absorbance values with the standard curve of AChE.

### 4.6. Western Blot Analysis of PC12 Cells

The cells were dissolved with RIPA lysis buffer (1× RIPA, 200 mM phenylmethylsulfonyl fluoride (PMSF), 100× phosphate inhibitor cocktail 2, 100× phosphate inhibitor cocktail 3, 100× protease). The protein concentration of the cell lysate was quantified (20 µg/mL) using a Pierce™ BCA Protein Assay Kit (Thermo Fisher Scientific, San Jose, CA, USA). It was loaded in 10% sodium dodecyl sulfate (SDS)-polyacrylamide gel electrophoresis (PAGE) and run at 200 V for 60 min. Samples were transferred by electrophoresis to polyvinylidene fluoride (PVDF) membranes at 20 V for 18 h in a 4 °C chamber. Membranes were blocked with 5% skim milk in a mixture of Tris-buffered saline and Tween 20 (TBST) for 1 h. Afterward, they were incubated with primary antibodies (1:1000 dilution of CREB, ERK1/2, p-CREB, p-ERK1/2 (Cell Signaling Technology, Beverly, MA, USA) and β-actin (Santa Cruz Biotechnology, Santa Cruz, CA, USA)) overnight at 4 °C. The membranes were washed three times with TBST and incubated with goat anti-mouse or mouse anti-rabbit antibodies (1:3000; Santa Cruz Biotechnology) for 45 min at room temperature. After washing three times, immunoreactivity was detected using an enhanced chemiluminescence detection system (GE Healthcare, Little Chalfont, UK). Bands were visualized using the ChemiDoc™ XRS+ system (Bio-Rad, Hercules, CA, USA). The intensity of each band was quantified using Image Lab^™^ software (Bio-Rad, Hercules, CA, USA).

### 4.7. Animals and Drug Treatments

Six-week-old male ICR mice (25–30 g) were purchased from the Orient Bio Co., Ltd. (Gyeonggi-do, Korea), and kept in the Dongguk University Laboratory Animal Research Center (Korea). All mice were housed under controlled conditions at 23 ± 3 °C, with a relative humidity of 50 ± 10%, room air changes 10–15 times/h, and a 12-h light/dark cycle (09:00–21:00). Food and water were provided ad libitum. The animals were used after 2 weeks of adaptation. All animal experiments in this study were approved by the Institutional Animal Care and Use Committee of Dongguk University, Seoul, Korea (approval no. 2015-DGU-052). To test the effects of CTFE on learning and memory function, the mice were randomly assigned to six treatment groups (*n* = 7). The ear of each mouse was marked with a permanent marker for easy identification. Water and CTFE were given daily via oral administration for 28 days. The volume of oral (p.o.) and intraperitoneal (i.p.) administration was 1 mL/100 g body weight (BW) of mice. BW was checked every day before oral administration. Treatments were administered to the six groups as follows:

Group 1: water, p.o. and normal saline, i.p. (vehicle control)
Group 2: water, p.o. + 2 mg/kg GAL, i.p. (positive control)
Group 3: water, p.o. + 1 mg/kg SCO, i.p. (negative control)
Group 4: 125 mg/kg/day CTFE, p.o. + 1 mg/kg SCO, i.p.
Group 5: 250 mg/kg/day CTFE, p.o. + 1 mg/kg SCO, i.p.
Group 6: 500 mg/kg/day CTFE, p.o. + 1 mg/kg SCO, i.p.

**Figure 7:**
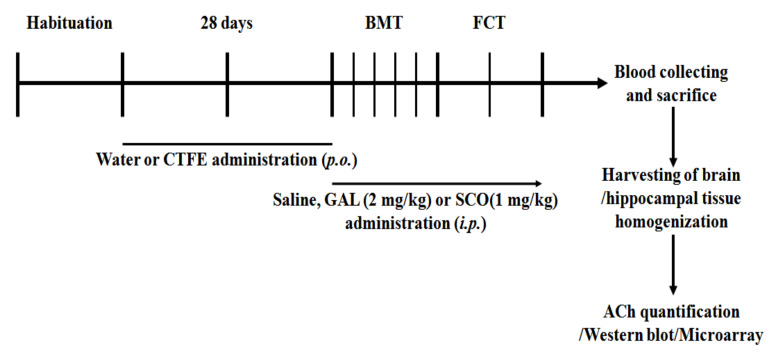
Experimental timeline of drug administrations and the behavioral test order in mice. After 2 weeks of habituation in the animal room, mice were administered saline or CTFE for 28 days once daily. All mice were sacrificed for hippocampal tissue collection. BMT: Barnes maze test, FCT: fear conditioning test, CTFE: *Cudrania tricuspidata* fruit extract, GAL: galantamine, SCO: scopolamine, ACh, acetylcholine.

For each behavioral test, except for the training phase in the Barnes maze and the hippocampal tissue analysis, mice were injected with normal saline, GAL, and SCO i.p. 30 min before each trial. An experimental timeline of drug administrations and the behavioral tests of mice are schematically shown (Figure 7).

### 4.8. Barnes Maze Test (BMT)

The Barnes maze test (BMT) is a dry-land maze test for spatial learning and memory using spatial cues [75]. Briefly, we prepared a maze consisting of a black platform (92 cm in diameter, 105 cm in height) with 20 holes (5 cm in diameter, 7.5 cm intervals between the holes) and a circumference of 2 cm (Jungdo B&P, Seoul, Korea). A black escape box (1064 cm) with bedding was placed under one of the holes (target/escape hole). Three simple colored-paper shapes (square, triangle, circle) were mounted around the room as visual cues. The test was performed in a room with a bright light (350 lux). When the mouse started exploring, a buzzer (85 dB) was activated to motivate the animal to find the target hole. At the end of each trial, the Barnes maze and escape box were cleaned three times with 70% ethanol, and fresh bedding was provided to prevent olfactory clues emanating from fecal boles and urine puddles. Each trial was recorded by a video camera and analyzed by behavior analysis software (EthoVision XT 9, Noldus, Wageningen, The Netherlands). The distance and latency to enter the escape box were measured during training phases (days 1–4) to determine the training level. In the probe phase (day 5), the maze was divided into quadrants, and the time in the target zone was measured to determine spatial learning [46].

### 4.9. Fear Conditioning Test (FCT)

The contextual and cued fear conditioning test (FCT) is a paradigm to study the biology of fear, anxiety, and memory [76]. Briefly, the mouse was exposed to conditional stimuli, such as tone, light, or place with an unconditional stimulus, like electrical foot shock. To perform the test, the mouse was placed in a conditioning chamber consisting of a grid floor able to transmit an electronic shock so that the mouse exhibited freezing behavior, speakers to provide tone as a cue, and an electric shock generator. The experimental conditions were under computer control. At the end of each phase, the mouse was returned to the home cage, the conditioning chamber was cleaned three times with 70% ethanol, and fresh bedding was provided to remove the fecal boles and urine puddles. Freezing behavior was defined as the absence of movement, excluding respiration, and was used as the index of fear [77]. Each phase was analyzed by behavior analysis software (EthoVision XT 9).

### 4.10. Blood Sampling and ACh Quantification

To assess the ACh concentration, blood was obtained from the retro-orbital plexus of mice under brief anesthesia using an ether-filled jar with a tight-fitting lid. A microhematocrit capillary tube was inserted into the corner of the eye socket underneath the eyeball of the anesthetized animal. The tip was directed toward the middle of the eye socket at a 45° angle [78]. Blood was collected, centrifuged (16,000 rpm at 4 °C for 5 min), and an aliquot of the separated serum was used for ACh quantification using a Choline/ACh Quantification Colorimetric/Fluorometric Kit (BioVision).

### 4.11. Hippocampal Tissue Homogenization

After all the behavior tests were completed, mice were sacrificed. Hippocampal tissues of sacrificed mice were homogenized in lysis buffer. After centrifugation (16,000 rpm at 4 °C for 15 min) of the homogenate, the protein content of the supernatant was quantified (50 µg/mL) by the BCA assay using a Pierce™ BCA Protein Assay Kit (Thermo Fisher Scientific).

### 4.12. AChE Activity in Hippocampal Tissue

Hippocampal tissue lysates were used to measure the AChE activity at 37 °C for 10–30 min, using the AChE Activity Colorimetric Assay Kit (BioVision). Absorbance at 570 nm was measured in a microplate ELISA reader (TECAN). The absorbance was compared with the AChE standard curve to determine the SCO- and CTFE-induced AChE activity.

### 4.13. Microarray Gene Expression Analysis

Microarray analysis was performed using the Affymetrix^®^ Mouse Gene 2.0 ST Array (e-Biogen Seoul, Korea). Total RNA was isolated from individual groups (vehicle, 1 mg/kg SCO, 500 mg/kg/day CTFE, and CTFE + SCO) using the RNeasy^®^ Mini Kit (Qiagen, Venlo, The Netherlands). The relative expression of genes was showed using hierarchical clustering analysis of Multiexperiment Viewer (MeV), v4.9.0.

### 4.14. Statistical Analysis

All analyses were performed using GraphPad Prism version 5.0 (GraphPad Software, Inc., San Diego, CA, USA). Data were expressed as mean ± SEM. Statistical significance was assessed by one-way and two-way analysis of variance (ANOVA), followed by Tukey’s multiple comparisons test at *p* < 0.05.
